# A pilot study: the impact of clinic-provided transportation on missed clinic visits and system costs among teenage mother–child dyads

**DOI:** 10.1057/s41599-022-01342-x

**Published:** 2022-09-16

**Authors:** Lao-Tzu Allan-Blitz, Aaida Samad, Kenya Homsley, Sojourna Ferguson, Simone Vais, Perry Nagin, Natalie Joseph

**Affiliations:** 1 Department of Pediatrics, Boston Medical Center, Boston, MA USA; 2 Division of Global Health Equity, Department of Medicine, Brigham and Women’s Hospital, Boston, MA USA; 3 Boston University School of Medicine, Boston, MA USA; 4 Department of Family and Community Medicine, University of California, San Francisco, San Francisco, CA USA; 5 Department of Adolescent Medicine, Boston Medical Center, Boston, MA USA

**Keywords:** Medical humanities, Science, technology and society

## Abstract

Transportation insecurity has profound impacts on the health and wellbeing of teenage parents and their children, who are at particularly high risk for missed clinic visits. In other settings, clinic-offered rideshare interventions have reduced the rates of missed visits. We conducted a one-arm pre-post time series analysis of missed visits before and after a pilot study rideshare intervention within a clinic specializing in the care of teenage parents and their children. We compared the number of missed visits during the study with the number during the preceding year (July 2019–March 2020), as well as the cost difference of missed visits, adjusting for inflation and clinic census. Of 153 rides scheduled, 106 (69.3%) were completed. Twenty-nine (29.9%) of 97 clinic visits were missed during the study period, compared to 145 (32.7%) of 443 comparison period visits (*p*-value = 0.59). The estimated cost difference of missed visits including intervention costs was a net savings of $90,830.32. However, the standardized cost difference was a net excess of $6.90 per clinic visit. We found no difference in rates of missed visits or costs, though likely impacted by the low census during the SARS-CoV-2 pandemic. Given the potential to improve health disparities exacerbated by the pandemic, further research is warranted into the impact and utility of clinic-offered rideshare interventions.

## Introduction

Lack of transportation is a major barrier to accessing healthcare (Hughes-Cromwick et al., 2005), driven in part by the distances patients must travel to reach the health facility, access to a vehicle, and requisite return transportation from the facility (Wolfe et al., 2020). As many as 5.8 million people in the United States may be unable to access healthcare due to lack of transportation (Syed et al., 2013). Specific populations at risk for transportation insecurity include older individuals, teenage parents, ethnic and racial minorities, those of lower socioeconomic status and those with lower levels of educational attainment (Wolfe et al., 2020; Ballantyne and Rosenbaum, 2017). Furthermore, children are particularly vulnerable to transportation limitations (Riportella-Muller et al., 1996; Giambruno et al., 1997) given their inherent dependence on adults. Infants of teen parents are at increased risk for accidents and early death, lower educational attainment, social and behavioral problems, and for becoming teenage parents themselves (Hofferth, 1987). In addition, teenage parents are more likely to be of lower socioeconomic status (Mangeli et al., 2017), and thus particularly susceptible to transportation insecurity. The Children’s Health Fund in 2010 noted 4% of US children had missed healthcare appointments due to lack of transportation, which increased to 9% in households with an average annual income below $50,000 (Grant et al., 2014). Those findings are supported by more recent estimates of 5% from a single institution, which also demonstrating improved pediatric follow-up rates after addressing transportation insecurity in 2022 (Hoffman et al., 2022).

The consequences of missed appointments are numerous, both to patients and institutions. Missing appointments leads to delays in necessary treatment and preventative care, which often translate to worsening rates of morbidity and mortality, particularly among children (Tin et al., 1998). Those who suffer from transportation insecurity are often more disenfranchised, thus missing an appointment further compounds the health impacts of that disenfranchisement as well as perpetuates the existing structural inequities among those populations. Additionally, missed appointments lead to exorbitant healthcare costs (Kheirkhah et al., 2016; Triemstra and Lowery, 2018), prompting clinics to discharge a patient from their clinic after a certain number of missed visits, which only exacerbates disparities in access to healthcare. Teenage parents and their children face numerous barriers to care (Mangeli et al., 2017; Hofferth, 1987) and may be at particularly high risk of both missed clinic appointments due to lack of transportation as well as the negative consequences of missing such appointments, as the lack of access to healthcare impacts both parent and child. As such, they may benefit uniquely from transportation assistance. Improving access to care among teenage parents and their children may be essential in facilitating a more equitable system of healthcare.

Non-emergent medical transportation services enable cost-effective financial supplementation for patient transport (Hughes-Cromwick et al., 2005). That cost effectiveness may be further augmented by novel technological innovations (Rochlin et al., 2019), which include collaboration with third-party rideshare vendors and have potentially broader patient accessibility. In 2016, Uber along with a digital platform that assists with non-emergent medical transportation collaborated to offer on-demand rides to a subset of individuals, resulting in missed-visit rates of 8%, compared to a non-emergent medical transpiration industry average of 25% (Surampudi, 2019). In 2018 Uber launched their own UberHealth platform that serves over 250 cities and over 100 healthcare organizations, resulting in a notable reduction in missed clinic appointments in several follow-up studies (Vais et al., 2020; Chaiyachati et al., 2018). Given the substantial health consequences of missed appointments among teenage parents and their children mentioned above, we implemented a rideshare intervention at a specialized clinic for teenage parent–child dyads in order to assess the impact on missed clinic visits, cost, and patient satisfaction.

## Methods

### Study setting

We conducted a pilot study at a single clinic, Teen and Tot, within Boston Medical Center, a tertiary care academic medical center and the largest safety net hospital in New England. The average rate of transportation insecurity (lack of available transportation to medical appointments) for patients of adult general medicine clinics at Boston Medical Center is 7.4%, while among pediatric clinics, the rate is approximately 11% (Buitron de la Vega et al., 2019). The Teen and Tot clinic serves pregnant adolescents up to the age of 20 years, as well as adolescent and young adult mothers and their children up to when the mother’s age reaches 23 years. The Teen and Tot clinic provides comprehensive care, support, and health education to nearly 600 mother–child dyads yearly through prenatal classes, parenting groups, and overall access to various medical services including routine pediatric and adolescent primary care. The population seen by the Teen and Tot clinic is composed predominantly of racial minorities (61% Black, 18% Hispanic, 14% White, and 7% other), and socioeconomically disadvantaged individuals (70% publicly insured, 30% privately insured).

### Description of intervention

We implemented a rideshare intervention to reduce missed-visit rates at Boston Medical Center’s Teen and Tot clinic from July 2020 through February 2021. Individuals with existing clinic appointments were screened during standard clinic appointment reminder calls, which occurred 1-2 days before the scheduled appointment. Eligibility was defined as existing patients within the Teen and Tot clinic network who had a cell phone, and who resided within 50 miles of the clinic. All eligible individuals were offered free transportation to their scheduled visits via the UberHealth platform. As of October 1st, based on positive responses to ongoing quality assessment surveys, we also offered return rides home from clinic. Rides were then scheduled by clinic staff using an existing rideshare account created by the clinic. The patient information required was a cell phone number and a pick-up address. All rideshare costs were incurred by the clinic, and included the market cost for each ride and no-show fees. All drivers had undergone driving record and criminal history checks as part of standard Uber procedures. Patients would automatically receive text message reminders about their ride for both the initial ride to the clinic as well as return ride reminders upon conclusion of their appointment. Patients were then surveyed after their appointments to assess ride satisfaction and concerns.

### Ethical approval and informed consent

The University and Medical Center Institutional Review Board exempted the study from review on the basis of its structure as a quality improvement project. All study procedures were done in accordance with relevant guidelines and regulations. Further, informed consent was deemed not required for this project by the institutional review boards. Hence, patients who verbally consented to receive rideshares were informed of study procedures and were eligible to participate in the intervention.

### Covariates and outcome measures

The primary independent variable of interest was the number of rideshares provided. Additional covariates of interest included process measures such as: number of patients offered a ride, number of completed rides provided, and complications arising as a result of the offered ride (e.g., missed ride and coordinator cancellations). We also collected the cost of each individual ride and canceled ride, as well as estimated administrative time required for ride scheduling, the average distance traveled, and average ride duration. Further, we collected general clinic measures, which included total number of clinic visits scheduled, number of missed visits, and visit type (annual exam or follow-up). Balancing measures included provider and staff satisfaction with the scheduling process, which were qualitatively collected during the bi-monthly review meetings of the progress of the pilot study, as well as patient satisfaction with rideshare experience, which was assessed using a likert scale survey (see Supplemental Table).

Primary outcome measures included missed clinic visits without cancellation and overall cost differences, comparing the time period of intervention (July 2020–February 2021) with the preceding year (July 2019–March 2020). The outcome of missed visits was defined as the proportion of missed clinic visits among the total number of clinic visits during the above time periods. We estimated a range of minimum and maximum cost differences, defined as the difference in cost of missed clinic visits pre-intervention and the cost of missed clinic visits during the intervention plus the overall cost of our intervention. The minimum cost difference was calculated as the maximum estimated cost of missed visits pre-intervention minus the minimum cost of missed visits inclusive of intervention costs post-intervention. The maximum cost difference was defined as the maximum cost pre-intervention minus the minimum cost inclusive of intervention costs post-intervention.

Formula for minimum cost difference:

$$\begin{array}{l}{\mathrm{Minimum}}\,{\mathrm{Cost}}\,{\mathrm{Difference}} = \left( {{\mathrm{Max}}.\,{\mathrm{Cost}}\,{\mathrm{of}}\,{\mathrm{Missed}}\,{\mathrm{Visits}}_{{\mathrm{Pre}} - {\mathrm{Intervention}}}} \right)\\ - \left( {{\mathrm{Min}}.\,{\mathrm{Cost}}\,{\mathrm{of}}\,{\mathrm{Missed}}\,{\mathrm{visits}}_{{\mathrm{Post}} - {\mathrm{Intervention}}} + {\mathrm{Intervention}}\,{\mathrm{Costs}}} \right)\end{array}$$

Our secondary outcome measure was patient satisfaction, assessed via phone surveys conducted after ride completion. Any rideshare associated adverse events were collected through patient satisfaction surveys or reported by patients to clinical coordinators.

### Data analysis

We conducted a pilot one-arm pre-post time series study comparing the rates of missed visits during the time period of our intervention with the rate of missed visits from the same time period in the preceding year. We used Chi square statistics to assess the difference in the proportion of missed visits between the two time periods.

For the cost analysis, we estimated the cost of our intervention, inclusive of direct expenditures on rides and administrative time (estimated to be a total of 15 h charged at a rate of $28/h). The administrative time estimate was based on the time required to schedule rides (estimated at 5 min per ride), and included an additional 2 min per canceled ride. That estimate also included time for 30-min bi-monthly meetings in which ongoing process measures were reviewed with study staff and clinic coordinators. Finally, we added an additional 30% of time to account for estimate variability. The cost of an annual physical exam or new patient visit was estimated to be $900.00, while the cost of a follow-up exam was estimated to be $300.00 based on interviews with clinic administrators. Prices for comparison period were adjusted for inflation using the consumer price index for July 2021 from July 2019, while prices for the study period were adjusted using the consumer price index for July 2021 from July 2020 (Bureau of Labor Statistics. Consumer Price Index-July 2021. Available at: https://www.bls.gov/news.release/pdf/cpi.pdf, Accessed August 23, 2021”). Those estimates were provided by Boston Medical Center clinic accountants and were inclusive of professional and facility expenses. We subsequently calculated the cost difference between the intervention period and the comparison period. Finally, we reported patient satisfaction results from the likert survey, with responses of “Agree” and “Strongly Agree” collapsed into positive responses, and all other responses collapsed into negative responses. All analyses were conducted using STATA 15.1 (StataCorp, College Station, TX). The data that support the findings of this study are available from Boston Medical Center but restrictions apply to the availability of these data, which were used under license for the current study, and so are not publicly available. Data are however available from the authors upon reasonable request and with permission of Boston Medical Center.

## Results

The total number of clinic appointments during the study period (July 2020– February 2021) was 97, 29 (29.9%) of which were missed visits without cancellation. For the period of July 2019–March 2020, the total number of clinic appointments was 443, of which 145 (32.7%) were missed visits without cancellation (*p*-value = 0.59). Table 1 shows the demographic characteristics of individuals presenting for care during each period.

**Table 1:** Demographic characteristics of Teen and Tot clinic patients July 2019–March 2020 vs. July 2020–February 2021.

	July 2019–March 2020		July 2020–February 2021		*p*-value
	No.	%	No.	%	
Total	151	100.0%	97	100.0%	
Age (years)					
14–20	41	27.2%	37	38.1%	0.18
21–23	68	45.0%	36	37.1%	
Race					
American Indian/Native American	1	0.8%	0	0.0%	0.22
Asian	2	1.6%	1	1.1%	
Black/African American	97	78.9%	60	68.2%	
Hispanic or Latino	13	10.6%	20	22.7%	
White	10	8.1%	7	8.0%	
Ethnicity					
Not Hispanic	109	72.7%	65	67.0%	0.34
Hispanic	41	27.3%	32	33.0%	
Highest education					
8th grade or less	6	4.1%	8	8.6%	0.06
Some High School	34	23.4%	34	36.6%	
Graduated High School/GED	55	37.9%	30	32.3%	
Some College/Vocational Program	37	25.5%	17	18.3%	
Graduated College/Post Graduate	13	9.0%	4	4.3%	

During the study period, a total of 153 rideshare trips were scheduled, of which 106 (69.3%) were completed, while 34 (22.2%) the user canceled and 13 (8.5%) the coordinator canceled. Data were not available on reasons for cancellations. Of the 97 clinic visits during the observation period, 56 (57.7%) were via rideshares. The remainder of the scheduled rideshares were for trips returning from the clinic. The average distance traveled was 4.4 miles each way, with an average ride duration of 13.2 min.

On average, each UberHealth ride cost $17.38 for completed trips, $5.82 for user-canceled rides, and $1.62 for coordinator-canceled trips. In total, we estimated the cost of our intervention to be $2,340.67 ($2,060.67 for the rides and $420.00 for the administrative time, charged at $28 per hour for an estimated total of 15 h of total work). The estimated total cost of the intervention and missed visits during the study period after adjusting for inflation from July 2020 to July 2021 was $26,321.00. The estimated total cost of missed visits during the during the comparison period, after adjusting for inflation from July 2019 to July 2021 was $117,151,32, resulting in a cost savings of $90,830.32 (Table 2).

**Table 2:** Cost estimate comparison of rideshare intervention with comparable timer period in the preceding year accounting for difference in missed clinic visits.

	Type of clinic visit	Cost of missed visits	Ride share costs	Administrative costs	Total costs adjusted for inflation	Crude cost difference	Standardized cost difference
Comparison period^a^	Physical exam	$99,900	N/A	N/A	$117,151.32	–	–
	Follow-up	$10,200.00					
Study period^b^	Physical exam	$20,700.00	$2060.67	$280.00	$26,321.00	$90,830.32	−$6.90
	Follow-up	$1800.00					

^a^Cost of missed clinic visits adjusted for inflation comparing July 2019 to July 2021.

^b^Costs of missed clinic visits adjusted for inflation comparing July 2020 to July 2021.

We further standardized the estimated costs of missed visits by the total number of clinic visits in each period in order to partially account for the impact of lower census due to the SARS-CoV-2 pandemic. The standardized intervention cost estimate was $271.35 per clinic visit, while the standardized cost estimate for the comparison period was $264.45 per clinic visit. The resulting cost difference was a net cost of $6.90 per clinic visit during the study period.

Sixty-four of 106 participants completed the post-ride survey. Of those, 60 individuals reported the ride increased their likelihood of presenting for their scheduled appointment and 43 (67.2%) reported they would have been unable to make their scheduled appointment otherwise. Among those surveyed who were offered return trips from clinic, 38 of 54 (70.4%) reported that the return trip was necessary for them to make their appointment. Importantly, 56 of the 64 individuals reported that they had no concerns regarding their health or safety during their ride; in fact, several reported feeling the rides offered were safer than public transportation. Six individuals raised safety concerns, three of whom reported challenges finding their ride, that the driver was late or never arrived. One reported that the driver was on the phone; one left personal belongings behind on the ride and had difficulty reaching the driver to coordinate retrieving their lost item. Two individuals did not respond to questions about safety concerns.

## Discussion

We implemented UberHealth ridesharing for pregnant teens as well as teenage mothers and their children in hopes of increasing access to healthcare for a high-risk population. Non-emergency medical transportation services have become essential pieces of healthcare equity in the United States given the risks to patients that are associated with missed visits and limited access to care (Tin et al., 1998; Hwang et al., 2015). Several studies have shown such interventions improve rates of missed visits (Surampudi, 2019; Vais et al., 2020; Chaiyachati et al., 2018) and are cost effective (Hughes-Cromwick et al., 2005; Rochlin et al., 2019). However, one controlled trial found no decrease in missed primary care appointments when uptake of the rideshare intervention was low (Chaiyachati et al., 2018). We found no significant difference in rates of missed clinic visits, and no clear benefit to system costs; however, those results are likely confounded by the reduced clinic census due to the severe acute respiratory syndrome coronavirus 2 (SARS-CoV-2) pandemic, as well as the small sample size of the pilot study.

Clinic appointment rates were considerably reduced across the country (Alexander et al., 2020), likely in part due to hesitancy to seek non-emergent care during the pandemic. Lower clinic census overall results in higher per-patient operating costs of both clinic visits and by extension our study intervention. Simultaneously, the implementation of virtual clinic visits likely further offset transportation insecurity and itself reduced the number of missed clinic visits (Drerup et al., 2021). Additionally complicating the interpretation of our results, prior work has demonstrated individuals view ridesharing services as carrying potentially higher risk for SARS-CoV-2 infection (Ozbilen et al., 2021), and thus may have avoided utilizing the offered services.

However, numerous studies have documented the disproportionate impact of SARS-CoV-2 on individuals of lower socioeconomic status (Chang et al., 2020; Hawkins, 2020), the principal population of the Teen and Tot clinic. Furthermore, the broad business closures consequent to the pandemic have also disproportionately impacted individuals of lower socioeconomic status (Usman et al., 2020; Nicola et al., 2020). Thus, individuals in our study who accepted the clinic-provided rideshare may have appreciated benefits from the provision of free ride to and from clinic beyond what was measurable.

Given those potential benefits, and the fact that the standardized cost difference in our study was relatively small, as well as prior literature supporting benefits of rideshare interventions (Vais et al., 2020; Vais et al., 2020; Chaiyachati et al., 2018), further research into other forms of rideshare programs is warranted as social distancing restrictions are eased. From our experience, other settings looking to implement rideshare interventions should consider providing further support, perhaps in the way of text messages or calls from the clinic, to remind patients of the scheduled ride in order to further reduce missed visits. Additionally, our post-survey results support the inclusion of return rides in future programs.

Our study had several additional limitations. Primarily, our sample size was limited, thus impacting the precision of our findings. Similarly, our study was from a single medical center, and a sub-population of individuals presenting to care, thus limiting generalizability. Further, the absence of a comparison group limits assertion of causation, thus our findings should be viewed as a pilot and further research controlling for other confounders is warranted. Finally, the costs estimates were imprecise, and we were unable to account for several factors including changes in timeliness as a result of our rideshare intervention, as cost savings from higher rates of on-time appointments may be significant. However, given the nature of the study was quality improvement, and that the population being discussed is a particularly vulnerable population, we feel those limitations do not negate the importance of our findings, and that our results warrant further expansion of clinic-offered rideshare interventions in other settings.

## Conclusion

We implemented a clinic-offered rideshare intervention serving teenage mothers and their children. Although we did not find a significant difference in rates of missed visits or clinic-incurred costs, those findings are likely heavily impacted by changing clinic dynamics during the SARS-CoV-2 pandemic. Given the potential benefits to patients and the healthcare system, as well as the potential to improve health disparities exacerbated by the pandemic, further research is warranted into the impact and utility of clinic-offered rideshare interventions.

## Supplementary information

Supplementary Information

## Data Availability

The data that support the findings of this study are available from Boston Medical Center but restrictions apply to the availability of these data, which were used under license for the current study, and so are not publicly available. Data are however available from the authors upon reasonable request and with permission of Boston Medical Center.
