# Supplementary material for: A pilot study: the impact of clinic-provided transportation on missed clinic visits and system costs among teenage mother–child dyads
Source: Humanit Soc Sci Commun. 2022 Sep 16;9(1):319. doi: 10.1057/s41599-022-01342-x (PMC9483513; doi:10.1057/s41599-022-01342-x)
Supplement: Supplementary file 1 — Supplementary Information [file 41599_2022_1342_MOESM1_ESM.docx]

|  | Strongly Disagree | Disagree | Neutral | Agree | Strongly Agree |
| --- | --- | --- | --- | --- | --- |
| I had concerns regarding my health or safety during my ride today |  |  |  |  |  |
| I had difficulty locating my Uber |  |  |  |  |  |
| I would have come to my appointment today if I had not been provided with an Uber ride |  |  |  |  |  |
| This ride made me more likely to come to my appointment |  |  |  |  |  |
| I would not have made it to the appointment if I did not have the Uber ride |  |  |  |  |  |
| I could only come if I had the round trip uber ride |  |  |  |  |  |
| Did you have any other question or concerns regarding your ride today? † |  |  |  |  |  |
| † This question was open ended and provided an unstructured opportunity for the participant to provide feedback | | | | | |

**Supplementary Table: Post Visit Survey of Patients Presenting to the Teen and Tots Clinic Who Traveled Via Clinic-Provided Rideshare**
